# Development of the Japanese version of the work-life integration scale: Focusing on the work environment in Japan

**DOI:** 10.1371/journal.pone.0356695

**Published:** 2026-09-11

**Authors:** Ayaka Uchiyama, Gen Takagi, Koubun Wakashima, Naoto Sunaga, Takahito Hoshino, Kengo Nonaka, Sourabh Kumar

**Affiliations:** 1 Graduate School of Education, Tohoku University, Sendai, Japan; 2 Faculty of Humanities and Social Sciences, Shizuoka University, Shizuoka, Japan; 3 Sunaga Research Institute Co. LTD, Tokyo, Japan; 4 Social Health Promotion Co. LTD, Tokyo, Japan; 5 Anand International College of Engineering, Jaipur, India; University of Missouri Columbia, UNITED STATES OF AMERICA

## Abstract

In Japan, long working hours have become normalized, sometimes leading to serious societal issues such as death from overwork (karoshi) and suicide due to overwork (karojisatsu). Although the importance of work-life balance has been increasingly emphasized as a countermeasure, the diversification of work styles driven by remote work and technology has made it harder to clearly separate work and personal life. In response, this study focuses on the concept of “Work-Life Integration” (WLI) and aims to develop a tool for quantitatively assessing the actual state of WLI. Furthermore, it seeks to clarify the relationship between employment conditions and WLI. This study employed a longitudinal design, and collected data using self-reported measures. The WLI scale was translated into Japanese, and an online survey was conducted to evaluate the construct validity, convergent validity, discriminant validity, internal consistency, and test-retest reliability of the Japanese version (WLIS-J). Data from 704 employed individuals aged 20 years and older living in Japan were analyzed. Confirmatory factor analysis indicated that the original four-factor model did not fit the WLIS-J. An exploratory factor analysis revealed a new three-factor structure, comprising “Interference between Work and Life,” “Work-Family Strain,” and “Work-Family Enrichment.” Convergent and discriminant validity were supported using five established scales measuring work-life enrichment, work-life conflict, job satisfaction, family satisfaction, and mental health. The Cronbach’s alpha for the overall scale was  .85, with subscales ranging from  .80 to  .89, indicating adequate internal consistency. Intraclass correlation coefficients ranged from  .60 to  .75, confirming temporal stability. Furthermore, hierarchical multiple regression analyses, with WLI as the dependent variable, revealed that WLI was predicted by personal, work-related, and family-related factors. These findings suggest that the WLIS-J is a useful instrument for assessing work-life integration among Japanese workers, with long working hours in particular emerging as a significant predictor of WLI.

## Introduction

In Japan, long working hours and the health issues associated with them have become serious social problems. According to the Organization for Economic Co-operation and Development (OECD), Japan’s annual working hours average 1,607 hours, which is considered moderate among OECD member countries [1]. However, this figure does not account for unrecorded working hours, such as unpaid overtime or work taken home [1]. Long working hours are closely linked to short sleep duration, chronic fatigue, and delayed recovery from exhaustion, all of which have been empirically shown to negatively affect both physical and mental health. In extreme cases, they can lead to death from overwork (karoshi) [2]. According to statistics from the National Police Agency and Cabinet Office, approximately 2,500 suicides each year are linked to work issues [3]. These findings suggest that Japan’s labor environment is characterized not by economic reasons, but by the link between long working hours and suicide. In response to these issues, the Japanese government has established institutional frameworks such as the Act Promoting Measures to Prevent Death and Injury from Overwork and the Outline for Measures to Prevent Death and Injury from Overwork. However, in Japan, there remains a deep-rooted cultural background where long working hours reflect diligence [4], making it difficult to drive sufficient improvement through policy alone.

### Differences in work styles between Japan and overseas

In contrast, Western countries tend to place greater emphasis on work–life balance (WLB) [5]. In the United Kingdom, legal frameworks supporting WLB—such as maternity and parental leave, expanded childcare services, and regulations on working hours—have been developed in response to growing awareness of workers’ health. Similarly, in France, family policy is prioritized, with financial support and childcare services playing a central role in helping working mothers balance employment and family responsibilities.

Traditionally, the concept of WLB promoted in Japan aimed for harmony between work and life, assuming a clear separation between an individual’s work and personal life [6]. Yet, both work and life are essential parts of life, and prioritizing one does not necessarily mean sacrificing the other. Rather, work and life are seen as mutually influencing each other. Therefore, the complete separation of work from life is unrealistic, and a new approach to WLB is required.

### Growing attention to work-life integration

In recent years, in addition to WLB, the concept of “Work-Life Integration” (WLI) has gained attention. WLI is defined as “the flexible and high-level integration of work within the company and personal life, aiming to create synergies through their fluid interaction, thereby achieving productivity and growth expansion, while enhancing the quality of life and achieving fulfillment and happiness” [7]. In WLI, the integration of work and life is expected to lead to a positive spillover, where experiences at work benefit personal life (family), and experiences in personal life (family) are returned to the work domain, creating a mutually beneficial dynamic. WLI represents a higher-order construct that encompasses both work–family conflict, which reflects negative interference between work and family roles, and work–family enrichment, which captures the mutually enhancing effects between these domains. In other words, WLI is seen as an important approach for protecting mental and physical health through flexible work styles and control over working hours. In Japan, where reforms in work styles are necessary, creating an environment that allows individuals to balance work and life effectively is essential to preventing tragedies such as death from overwork (karoshi) and promoting a sustainable work environment. Existing research on WLI has primarily focused on its conceptual structure and the factors that define it. The conceptual structure of WLI consists of the following three elements [8]: First, there is bidirectional interference between work and family (work-to-family and family-to-work interference). Within the domains of work and family, there are physical, temporal, and behavioral boundaries, and individuals are required to fulfill roles in both areas. However, with modern and diverse work styles, these boundaries are becoming increasingly blurred, leading to an interference between work and family roles. If this interference is managed properly, it can lead to increased productivity and performance in both domains. However, if a balance cannot be maintained, efficiency and commitment may be negatively affected. Therefore, interference between work and family may either cause conflict or lead to fulfillment [9].

Second, work-family strain refers to the stress that arises from interference between work and family roles. When such interference becomes difficult to manage, it affects responsibilities in both domains and increases stress. For instance, working from home may reduce time for family activities and personal relaxation, thereby interfering with one’s personal life. Similarly, when family matters interfere with work during office hours, they can reduce work efficiency and elevate stress. Without proper management, this interference makes it difficult to maintain a balance between work and family, leading to a vicious cycle of stress accumulation.

Third, work–family enrichment refers to the positive effects that arise when work and family interact harmoniously. Experiences in the family domain can enhance work, and success at work can enrich family life. As a result, individuals experience improved mental and physical health and greater productivity [10].

Thus, WLI has been associated with satisfaction in both work and life, as well as with better mental health. Furthermore, WLI may have positive impacts not only on individual workers, but also on organizations. In addition, research on WLI has led to the development of measurement scales for assessing these concepts [11]. These scales consist of four factors: “Family to Work Interference,” “Work to Family Interference,” “Work-family Strain,” and “Work-family Enrichment,” with their reliability and validity having been confirmed [11].

Furthermore, previous WLI research has explored the determinants of workers’ WLI from three perspectives: personal factors, workplace and job factors, and family factors.

First, personal factors relate to workers’ basic attributes. Research shows that women are more likely than men to experience role conflict between work and family, although this gender gap is smaller in countries with more advanced gender equality [12]. However, a meta-analysis found a weak correlation between gender differences and work-family interference [13], suggesting that gender alone does not determine WLI. Instead, it is likely that work styles that promote WLI play a more critical role.

Second, workplace and job factors include working hours, work arrangements, and job characteristics. For example, a study in the United States pointed out that physicians, who are forced to work longer hours than other workers, struggle more with WLI [14]. Additionally, Byron et al. found that job involvement, job stress, working hours, social support from colleagues and supervisors, and flexible work arrangements all relate to work-family interference [13]. This finding is also supported by Munjal and Anooja [8], who identified that flexible work arrangements, such as part-time work and flextime, are positively associated with WLI. These findings suggest that workplace and job-related factors play a significant role in determining workers’ ability to achieve WLI.

Third, family factors include marital status, employment status of partners/spouses, and the number and age of children. A meta-analysis by Byron et al. on the impact of these factors on WLI found that workers with children experienced more work-related stress than those without. Furthermore, being single was positively related to bidirectional work-family interference [13]. Moreover, Japan is a super-aged society, and an increase in “business carers” balancing work with caregiving responsibilities is expected [15]. Considering this social trend, the burden of caregiving at home is likely to significantly affect WLI. Although family factors have been suggested to influence workers’ WLI, the findings are inconsistent [12]. Given Japan’s unique work styles, further detailed examination is required.

### Purpose of the study

Previous research on WLI has several remaining research gaps. First, there is a lack of international comparative research. While an English version of the WLI scale has been developed [11], differences in working styles between Japan and other countries highlight the need for a tool that appropriately captures the concept of WLI within the Japanese context. Second, the specific factors related to employment conditions that determine workers’ WLI remain unclear. Previous studies have suggested that personal, job, and family factors are related; however, it has not been confirmed whether similar findings hold true in Japan.

Therefore, this study has two objectives. First, it aims to translate the WLI scale [11] into Japanese and evaluate its reliability and validity within the Japanese context. Second, this study examines the factors of workers’ employment conditions that determine WLI in Japan. Specifically, this study explores how personal, job, and family factors influence WLI, while accounting for their interrelationships and assessing the magnitude of their direct impacts.

## Methods

This study was conducted in two stages. The first stage involved a preliminary survey that focused on translating the WLI scale into Japanese, following the International Society for Pharmacoeconomics and Outcomes Research (ISPOR) guidelines [16]. The second stage consisted of a full survey, which assessed the reliability and validity of the Work life integration scale Japanese version (WLIS-J) using an online survey, based on the Consensus-based Standards for Selection of Health Measurement Instruments (COSMIN) checklist [17]. All procedures were performed in accordance with the Declaration of Helsinki. Ethical approval was obtained from the ethics committee of ○○ University (Approval No. 24-1-070).

Data were collected in two waves with a three-week interval to assess test–retest reliability [18], with surveys administered on January 14 and February 4, 2025. Prior to participation, all prospective participants were provided with a detailed explanation of the study’s purpose and procedures via the online survey platform. Informed consent was obtained electronically; only individuals who explicitly agreed to participate were able to access and complete the questionnaire. All participants were employed adults aged between 21 and 69 years; therefore, the study did not involve any minors.

### Preliminary study (Development of the Japanese version of Work-Life Integration scale)

The WLI scale was translated following ISPOR guidelines [16]. First, permission to create the Japanese version was obtained from the original author of the WLI scale, Professor Sourabh Kumar. A forward translation was then conducted by a translation company, after which, the author and translation company jointly refined the Japanese expressions of the items. A back translation was then performed by the translation company. The back-translated scale was submitted to the original author to examine any discrepancies between the original and Japanese versions and to confirm whether the meaning of the original was maintained. The expressions of instructions and options were also reviewed. As a result, the original author judged that there were no issues, and the items in the Japanese version were finalized. The completed Japanese version of the scale consists of 17 items, identical to the original version, including: “Family to Work Interference” (4 items), “Work to Family Interference” (4 items), “Work-family Strain” (6 items), and “Work-family Enrichment” (3 items). These 17 items were designated as the provisional Japanese Work-Life Integration Scale (WLIS-J). Each item is rated on a 7-point Likert scale, ranging from 1 (“not at all applicable”) to 7 (“most applicable”). The full Japanese version of the Work-life Integration scale is provided as Supporting Information (S1 Appendix Japanese version of the Work-life Integration Scale and S2 Appendix English Translation.) and is available under the Creative Commons Attribution 4.0 International License (CC BY 4.0).

### Main survey

#### Procedure.

The initial measurement focused on collecting demographic information, WLIS-J, work-life enrichment, work-family conflict, job satisfaction, family satisfaction, mental health, and items related to the identification of unreliable respondents.

Demographic variables included age, gender, highest educational qualification, and current employment status. Specifically, information was collected on job position, number of employees in the organization, years of service, average weekly workdays, average daily working hours, weekly remote working hours, overtime hours (per month), daily commuting time (in minutes), weekly rest days, and household income. Work characteristics were also assessed, including the need for communication with internal and external stakeholders, physical and mental workloads, job complexity, and the frequency of irregular tasks. These work characteristics were rated on a 5-point scale ranging from 1 (low) to 5 (high).

Information regarding career history, marital status, and family composition was collected. Participants were asked about their marital status (married, unmarried, separated, or divorced); for married participants, further details were collected regarding their spouse’s employment status and average daily working hours. Family related data included the number of children living in the household, the age of the youngest child, and caregiving responsibilities. Specifically, participants were asked whether they were providing home-based caregiving (yes or no), the duration of caregiving (in months), and the average caregiving hours per day for those engaged in caregiving.

WLIS-J: The items developed in the preliminary survey (17 items across 4 factors, rated on a 7-point scale) were used.

Work-Family Enrichment (WFE): The Japanese version of the Work-Family Enrichment (WFE) Scale [19], based on the original scale [20], was used. This scale consists of 6 factors and 18 items, and its reliability and validity have been confirmed [19]. The items assess “development from work to family,” “emotion from work to family,” “capital from work to family,” “development from family to work,” “emotion from family to work,” and “efficiency from family to work,” with responses rated on a 5-point Likert scale ranging from 1 (strongly disagree) to 5 (strongly agree). This scale was used to confirm the validity of WLIS-J.

Work-Family Conflict (WFC). The Japanese version of the multidimensional Work-Family Conflict Scale [21], based on the original scale [22], was used. This scale consists of 6 factors and 18 items, and its reliability and validity have been confirmed [21]. The items assess “time-based work→family conflict,” “time-based family→work conflict,” “stress response-based work→family conflict,” “stress response-based family→work conflict,” “behavior-based work→family conflict,” and “behavior-based family→work conflict,” with responses rated on a 5-point Likert scale ranging from 1 (strongly disagree) to 5 (strongly agree). This scale was used to confirm the validity of WLIS-J.

Job Satisfaction: Participants were asked to rate the following items on a 5-point scale ranging from 1 (strongly disagree) to 5 (strongly agree): “Overall, I am satisfied with my job,” “Overall, I like working at my workplace,” and “Overall, I do not enjoy my job (reverse-coded item)” [18]. This measure was used to confirm the validity of WLIS-J.

Family Satisfaction: Participants were asked to rate the following items on a 5-point scale ranging from 1 (strongly disagree) to 5 (strongly agree): “Overall, I am satisfied with the time spent with my family,” “Overall, I enjoy spending time with my family,” and “Overall, I do not enjoy spending time with my family (reverse-coded item)” [18]. This measure was used to confirm the validity of WLIS-J.

Mental Health: The Japanese version of the Kessler Psychological Distress Scale (K6) [23] was used. The K6 screens for mood disorders (such as major depression and dysthymia) and anxiety disorders (including panic disorder, agoraphobia, social anxiety, generalized anxiety disorder, and PTSD) over the past 12 months. Responses were rated on a 5-point Likert scale ranging from 1 (none of the time) to 5 (all of the time) [24]. This scale was used to confirm the validity of WLIS-J.

Identification of Unreliable Respondents: The Instructional Manipulation Check (IMC) task was used [25]. The instructions included a directive asking participants to skip a question without selecting any option and proceed by clicking the “next” button. This was used to identify unreliable respondents.

### Statistical analysis

Statistical analysis was conducted using SPSS for Windows version 29.0, and Amos for Windows version 29.0. Data from the first time point were used to assess the factor structure, and both time points were used to evaluate test-retest reliability. Only responses that met the IMC condition were included in the analysis.

First, to verify the factor structure of the original scale, confirmatory factor analysis (CFA) was performed on the WLIS-J. Goodness-of-fit indices and factor loadings were examined based on the following thresholds: CFI ≥ .90, RMSEA ≤ .08, and factor loadings ≥ .32 [26]. If the model did not meet the established guidelines, exploratory factor analysis (EFA) was conducted to reconsider the factor structure. Subsequently, CFA was performed again to assess the model’s fit. If a factor structure different from the original was identified, the factor structure and items were compared with the original scale. The validity of the content was then reviewed by multiple researchers specializing in clinical psychology to ensure its appropriateness.

Test–retest reliability and internal consistency were evaluated using intraclass correlation coefficients (ICCs) and Cronbach’s alpha, respectively. These analyses provide evidence of the psychometric reliability of the WLIS-J. Second, Hierarchical regression analyses were conducted to examine the predictive contribution of personal, work-related, and family-related factors to WLI. To examine the contribution of different sets of predictors to WLI, hierarchical multiple regression analyses were conducted with each subscale of the WLIS-J as the dependent variable. This analytic strategy was chosen because hierarchical regression allows for the assessment of whether changes in R² between steps are statistically significant, thereby enabling the evaluation of the incremental contribution of each set of predictors.

## Results

### Selection of participants for analysis

A survey was conducted with a sample of 1,000 employed individuals aged 20–69. Of these, 293 participants were excluded from the analysis because they provided unreliable responses based on the IMC condition, and three participants were excluded for not meeting the eligibility criteria. The final sample for analysis consisted of 704 participants (363 males, 341 females) with a mean age of 46.79 years (SD = 13.27, range 21–69 years). Baseline demographic characteristics of the respondents are presented in Table 1.

**Table 1:** Demographic characteristics of survey participants.

Category	First Survey (n = 704)	Second Survey (n = 283)
**Age (years)**		
20-29	114 (16.2%)	33 (11.7%)
30-39	119 (16.9%)	47 (16.6%)
40-49	144 (20.5%)	74 (26.1%)
50-59	164 (23.3%)	73 (25.8%)
60-69	163 (23.2%)	56 (19.8%)
**Gender**		
Male	363 (51.6%)	169 (59.7%)
Female	341 (48.4%)	114 (40.3%)
**Marital Status**		
Married	318 (45.2%)	128 (45.2%)
Unmarried	331 (47.0%)	132 (46.6%)
Separated/Divorced	55 (7.8%)	23 (8.1%)
**Spouse’s Employment Status**		
Full-time Regular Employee	162 (23.0%)	60 (21.2%)
Permanent Full-time (Part-time)	5 (0.7%)	14 (4.9%)
Other Employment Status	24 (3.4%)	7 (2.5%)
Not Working	76 (10.8%)	35 (12.4%)
**Household Income**		
Less than 1 million yen	22 (3.1%)	7 (2.5%)
1 million-2 million yen	20 (2.8%)	13 (4.6%)
2 million-3 million yen	64 (9.1%)	23 (8.1%)
3 million-4 million yen	81 (11.5%)	28 (9.9%)
4 million-5 million yen	86 (12.2%)	31 (11.0%)
5 million-6 million yen	81 (11.5%)	31 (11.0%)
6 million-7 million yen	54 (7.7%)	15 (5.3%)
7 million-8 million yen	65 (9.2%)	30 (10.6%)
8 million-9 million yen	46 (6.5%)	20 (7.1%)
9 million-10 million yen	47 (6.7%)	17 (6.0%)
10 million-12 million yen	61 (8.7%)	27 (9.5%)
12 million-15 million yen	43 (6.1%)	20 (7.1%)
15 million-18 million yen	10 (1.4%)	5 (1.8%)
18 million-20 million yen	7 (1.0%)	5 (1.8%)
20 million and above	17 (2.4%)	11 (3.9%)
**Household Structure**		
Single-person Household	192 (27.3%)	82 (29.0%)
Couple-only Household	134 (19.0%)	50 (17.7%)
Couple with Unmarried Children	218 (31.0%)	91 (32.2%)
Single Parent with Unmarried Children	53 (7.5%)	16 (5.7%)
Three-generation Household	44 (6.3%)	17 (6.0%)
Other Household Types	63 (8.9%)	27 (9.5%)
**Children in Household**		
No children	506 (71.9%)	203 (71.7%)
1 child	95 (13.5%)	37 (13.1%)
2 children	77 (10.9%)	32 (11.3%)
3 children	20 (2.8%)	9 (3.2%)
4 or more children	6 (0.9%)	2 (0.7%)
**Home Caregiver Status**		
Yes	41 (5.8%)	16 (5.7%)
No	663 (94.2%)	267 (94.3%)

### Verification of factor structure

#### Structural validity.

A CFA was conducted using the four-factor model outlined in the original study. The results indicated that the model did not demonstrate an adequate fit, with the following fit indices: CFI = .731, TLI = .693, RMSEA = .140 (90% Confidence Interval [CI] = 0.134–0.146), and SRMR = .2648. Given the insufficient fit, an EFA was subsequently performed.

To verify the adequacy of the sample size, both the Kaiser-Meyer-Olkin (KMO) test and Bartlett’s test of sphericity were conducted. The KMO value was 0.91, and Bartlett’s test of sphericity yielded a significant result (p < .001), confirming the appropriateness of conducting factor analysis.

Initially, floor effects were observed in items 1, 3, 4, 5, 7, 8, and 14 of the 17 items in the original version. However, because these items were deemed essential for explaining the concept of WLI, they were retained for further analysis. EFA was conducted on all 17 items using maximum likelihood estimation and promax rotation. The eigenvalues for the first three factors were 5.991, 2.220, and 1.244, respectively. The scree plot and cumulative variance explained (55.616%) supported a three-factor structure, which was further validated by the interpretability of the factors. Items were retained based on a minimum factor loading of  .35 on a single factor. The final set consisted of 17 items across three factors (Table 2).

**Table 2:** Structure coefficients for WLIS-J (n = 704).

Item	Factor 1	Factor 2	Factor 3
**F1: Interference between Work and Family (α = .88)**			
4. I often interrupt my work at the workplace to deal with family concerns.	0.835	−0.056	−0.008
1. I occasionally perform household tasks while at work.	0.804	−0.087	0.084
3. My family contacts me during working hours.	0.744	−0.112	0.037
8. I stop doing household chores to resolve work-related issues.	0.717	0.079	0.038
5. I bring work home with me.	0.632	0.033	−0.018
7. I almost always receive work-related calls at home after work.	0.628	0.016	0.092
2. I am concerned about my family while I am at work.	0.610	0.109	−0.010
14. I feel separated from my family due to my work.	0.609	0.100	−0.137
**F2: Work Family Strain (α = .89)**			
11. I feel mentally exhausted when I return home after finishing work.	−0.172	0.995	0.046
10. I feel physically exhausted when I return home after finishing work.	−0.153	0.991	0.117
13. I feel like I have to rush or stay constantly busy to manage work and household tasks every day.	0.147	0.630	−0.012
9. I feel that I am taking on more work than I can handle.	0.247	0.571	−0.114
6. I always think about work-related problems when I am at home.	0.244	0.556	−0.018
12. I feel like I don’t have enough time for myself.	0.114	0.544	−0.127
**F3: Work Family Enrichment (α = .80)**			
15. I am able to balance work and family in my current job.	−0.027	0.131	0.828
16. I have enough time for both work and personal life.	0.060	−0.032	0.755
17. I am satisfied with the schedule of my current job.	0.097	−0.076	0.707
Proportion of variance	35.24	13.06	7.32
Cronbach’s α (subscale)	0.88	0.89	0.80

*Note:* Extraction method: Maximum likelihood estimation. Rotation method: Promax rotation.

Bold font shows factor loadings greater than 0.35.

Next, CFA was performed on the three-factor, 17-item structure obtained from the EFA. The results showed a good model fit, with the following indices: χ² (47) = 30.533, p = .970, CFI = 1.000, TLI = 1.008, RMSEA = 0.00 (90% CI [0.000, 0.000]), and SRMR = .0111. These values indicated that the model provided an excellent fit to the data. Accordingly, the three-factor model was adopted in this study. Following this procedure, the final three-factor structure of WLIS-J was determined.

Descriptive statistics for the WLIS-J and other scales are presented in Table 3. The reliability coefficients for the WLIS-J were α = .85 for the entire scale, α = .88 for the first factor, α = .89 for the second factor, and α = .80 for the third factor. Given the sufficient internal consistency of each subfactor, the mean score of each factor was calculated and used as the representative factor scores in subsequent analyses.

**Table 3:** Mean, standard deviations and correlation among variables (n = 704).

Scale	Mean	SD	IWF	WFS	WFE
IWF (α = .88)	2.297	1.106	–		
WFS (α = .89)	3.33	1.342	.53**	–	
WFE (α = .80)	4.073	1.331	−.09*	−.33**	–
WFE Scale (α = .98)	3.033	0.911	.23**	0.03	.60**
WFC Scale (α = .96)	2.416	0.828	.64**	.66**	−.25**
Job Satisfaction (α = .80)	3.122	0.986	−0.02	−.28**	.39**
Family Satisfaction (α = .77)	3.564	0.928	−0.01	−0.07	.26**
K6 (α = .94)	1.855	0.987	.28**	.47**	−.26**

Note: *p < .05, **p < .01.

The first factor, consisting of items related to performing household tasks during work and bringing work home, was named “Interference between Work and Family (IWF).” The second factor, which included items concerning mental and physical fatigue or stress caused by work and family demands, was named “Work Family Strain (WFS),” following the original version. The third factor, consisting of items related to maintaining separate time for both work and family, and being able to balance the two domains, was named “Work Family Enrichment (WFE),” again following the original version.

The reliability, basic statistics, and correlations were first calculated using the available data without missing values (n = 682; 355 males, 327 females; mean age = 46.75 years; SD = 13.19; range = 21–69 years). The means and standard deviations of each variable are presented in Table 4.

**Table 4:** Means and standard deviations among variables (n = 682).

	Male			Female		
Item	N	Mean	SD	N	Mean	SD
1. Age	355	47.18	13.28	327	46.29	13.09
2. Years of Employment (Tenure)	355	16.73	11.94	327	13.56	11.01
3. Average Work Hours per Day (hours/day)	355	8.35	1.96	327	7.56	1.63
4. Recent Work from Home Hours (Past Week)	355	7.82	16.35	327	6.02	12.25
5. Commute Time (One-way, minutes)	355	35.83	27.94	327	31.8	24.74
6. Average Working Days per Week	355	5.03	0.83	327	4.95	0.76
7. Number of Holidays	355	1.95	0.61	327	2.04	0.73
8. Overtime Hours	355	5.9	11.11	327	2.94	6.03
9. Household Income (in thousand yen)	355	678.85	418.87	327	556.78	380.39
10. Need for Communication with Colleagues	355	3.45	1.25	327	3.44	1.27
11. Need for Communication with External People	355	3.36	1.28	327	3.2	1.34
12. Physical Burden	355	2.86	1.17	327	2.57	1.23
13. Mental Burden	355	3.45	1.07	327	3.41	1.14
14. Job Complexity	355	3.42	1.04	327	3.19	1.11
15. Irregular Work Amount	355	3.28	1.05	327	3.13	1.13
16. Number of Job Changes	355	2.56	1.8	327	3.15	2.01
17. Spouse/Partner’s Average Work Hours per Day	176	4.15	3.6	131	8.12	3.08
18. Number of Children Living with You	120	1.84	1.16	73	1.62	0.98
19. Age of the Youngest Child Living with You	81	7.32	6	49	6.98	5.94
20. Duration of Home-based Care (Months)	20	44.6	61.59	20	42	51.84
21. Average Caregiving Hours per Day	20	2.7	1.42	20	3.45	3.12

Note: *p < .05, **p < .01.

Next, a correlation analysis was conducted between WLIS-J and other variables (Table 5, 6).

**Table 5:** Correlations between WLIS-J subscales and related variables among male participants.

Item	IWF	WFS	WFE
1. IWF	—		
2. WFS	.60**	—	
3. WFE	−.03	−.26**	—
4. WFE Scale	.26**	.14**	.34**
5. WFC Scale	.68**	.68**	−.18**
6. Job Satisfaction	.04	−.20**	.43**
7. Family Satisfaction	−.05	−.02	.21**
8. K6	.29**	.50**	−.21**
9. Age	−.19**	−.12*	−.08
10. Years of Employment	−.10	−.04	−.09
11. Average Work Hours per Day	−.01	.24**	−.21**
12. Recent Work from Home Hours	.16**	.01	.05
13. Commute Time	.04	.16**	−.03
14. Average Working Days per Week	−.09	−.07	−.12*
15. Number of Holidays	.00	−.04	.08
16. Overtime Hours	.22**	.25**	−.15**
17.Household Income	.10	.10	.05
18. Need for Communication with Colleagues	.09	.24**	−.07
19. Need for Communication with External People	.15**	.23**	−.19**
20. Physical Workload	.20**	.37**	−.22**
21. Mental Stress	.14**	.42**	−.28**
22. Job Complexity	.13*	.28**	−.08
23. Frequency of Irregular Tasks	.15**	.35**	−.23**
24. Number of Job Changes	.08	.11*	−.08
25. Spouse/Partner’s Average Work Hours per Day	.05	.04	−.04
26. Number of Children Living with You	.15	.26**	.00
27. Age of the Youngest Child Living with You	−.30**	−.16	−.12
28. Duration of Home-based Care	.04	.04	−.35
29. Average Caregiving Hours per Day	.58**	.22	.31

Note: *p < .05, **p < .01.

**Table 6:** Correlations between WLIS-J subscales and related variables among female participants.

Item	IWF	WFS	WFE
1. IWF	—		
2. WFS	.48**	—	
3. WFE	−.17**	−.39**	—
4. WFE Scale	.17**	.00	.30**
5. WFC Scale	.59**	.64**	−.30**
6. Job Satisfaction	−.08	−.33**	.35**
7. Family Satisfaction	−.01	−.10	.30**
8. K6	.33**	.43**	−.30**
9. Age	−.09	−.10	.11*
10. Years of Employment	−.04	−.02	−.04
11. Average Work Hours per Day	−.01	.24**	−.31**
12. Recent Work from Home Hours	.24**	.02	−.05
13. Commute Time	.04	.16**	−.07
14. Average Working Days per Week	.01	.01	−.14**
15. Number of Holidays	−.11	−.14**	.13*
16. Overtime Hours	.18**	.23**	−.19**
17.Household Income	.00	.03	−.03
18. Need for Communication with Colleagues	.04	.25**	−.04
19. Need for Communication with External People	.13*	.22**	−.08
20. Physical Workload	.23**	.45**	−.25**
21. Mental Stress	.26**	.64**	−.27**
22. Job Complexity	.27**	.48**	−.20**
23. Frequency of Irregular Tasks	.26**	.45**	−.23**
24. Number of Job Changes	−.10	−.12*	.04
25. Spouse/Partner’s Average Work Hours per Day	−.01	.03	−.15
26. Number of Children Living with You	.00	−.05	−.01
27. Age of the Youngest Child Living with You	.02	−.09	.24
28. Duration of Home-based Care	.04	−.14	.15
29. Average Caregiving Hours per Day	.20	.14	−.57**

Note: *p < .05, **p < .01.

### Examination of WLIS-J prescriptive factors

To identify the predictors of the WLIS-J, a hierarchical multiple regression analysis was conducted using the WLIS-J subfactor scores as the dependent variables. To account for multiple testing across the three hierarchical regression models, the Holm–Bonferroni correction was applied to adjust the significance level. Consequently, the adjusted alpha level for statistical significance was set at 0.0167 (i.e., 0.05 divided by three tests). Power analysis was conducted based on this most conservative alpha value.

Gender was treated as a personal factor and was entered at Step 1 (Male = 1, Female = 0) to help elucidate the sociological characteristics of WLI in the Japanese context. At Step 2, work-related factors were entered, including tenure (years), working hours (hours/day), telecommuting hours (last week), commute time (minutes), working days per week, overtime hours, and job changes (number). At Step 3, family-related factors were entered, including household income (thousand yen), marital status (Married = 1, Unmarried/Divorced = 0), presence of minor children (Yes = 1, No = 0), and caregiving at home (Yes = 1, No = 0).

IWF: After controlling for gender, tenure was negatively associated with IWF, whereas telecommuting hours, overtime hours, presence of cohabiting children, and in-home caregiving were positively associated with IWF. The R² increases from Step 1 to Step 2 (ΔR² = 0.086, F = 9.352, p < .001) and from Step 2 to Step 3 (ΔR² = 0.047, F = 9.443, p < .001) were both significant, indicating that work-related and family-related factors significantly influenced IWF.

WFS: Similarly, after controlling for gender, tenure, telecommuting hours, overtime hours, presence of cohabiting children, and in-home caregiving were significant predictors of WFS. The R² increases from Step 1 to Step 2 (ΔR² = 0.107, F = 11.490, p < .001) and from Step 2 to Step 3 (ΔR² = 0.014, F = 2.700, p = .030) were significant, suggesting that both work- and family-related factors play important roles in WFS.

WFE: After controlling for gender, tenure, working hours, weekly working days, and overtime hours were negatively associated with WFE, whereas household income was positively associated. The R² increase from Step 1 to Step 2 was significant (ΔR² = 0.091, F = 9.629, p < .001), but the increase from Step 2 to Step 3 was not (ΔR² = 0.007, F = 1.281, p = .276), indicating that family-related factors did not significantly improve the explanatory power of the WFE model. These results suggest that work-related factors reduce WFE, while higher household income contributes positively. However, the increase in R² from Steps 2–3 was not significant, indicating that the addition of family related factors did not significantly enhance the explanatory power of the WFE model. All results are summarized in Table 7.

**Table 7:** The result of hierarchical multiple regression analysis.

	Criterion Variables																				
Predictor	IWF							WFS							WFE						
	*β*	*SE*	*p*	95%CI		adj *R*^2^	*△R* ^2^	*β*	*SE*	*p*	95%CI		adj *R*^2^	*△R* ^2^	*β*	*SE*	*p*	95%CI		adj *R*^2^	*△R* ^2^
Step1						.031	.032						-.001	.000						-.001	.000
Gender	.180	.084	<.001	.235	.564			-.016	.103	.681	-.244	.159			-.012	.102	.755	-.232	.169		
Step2						.108	.086						.096	.107						.080	.091
Gender	.158	.084	<.001	.186	.518			-.088	.102	.022	-.436	-.034			.063	.103	.105	-.035	.368		
Tenure	-.104	.004	.006	-.017	-.003			-.039	.004	.298	-.013	.004			-.080	.004	.036	-.018	-.001		
Working Hours	-.050	.023	.193	-.075	.015			.190	.028	<.001	.083	.192			-.231	.028	<.001	-.221	-.111		
Telecommuting Hours	.179	.003	<.001	.008	.019			.026	.003	.484	-.004	.009			.006	.003	.871	-.006	.007		
Commute Time	.032	.002	.391	-.002	.004			.108	.002	.005	.002	.009			-.014	.002	.715	-.004	.003		
Working Days per Week	-.064	.051	.081	-.190	.011			-.058	.062	.116	-.219	.024			-.104	.062	.006	-.295	-.051		
Overtime Hours	.208	.005	<.001	.016	.034			.185	.006	<.001	.016	.038			-.093	.006	.017	-.025	-.002		
Job Changes	-.015	.022	.682	-.051	.034			.001	.026	.985	-.051	.052			-.043	.026	.265	-.081	.022		
Step3						.150	.047						.105	.014						.082	.007
Gender	.150	.083	<.001	.171	.498			-.084	.103	.028	-.427	-.024			.059	.104	.127	-.045	.361		
Tenure	-.126	.004	.001	-.019	-.005			-.037	.004	.338	-.013	.004			-.087	.004	.025	-.019	-.001		
Working Hours	-.044	.023	.240	-.071	.018			.192	.028	<.001	.084	.194			-.239	.028	<.001	-.227	-.116		
Telecommuting Hours	.173	.003	<.001	.008	.019			.018	.003	.635	-.005	.008			.002	.003	.952	-.007	.007		
Commute Time	.023	.002	.528	-.002	.004			.106	.002	.006	.002	.009			-.022	.002	.576	-.005	.003		
Working Days per Week	-.059	.050	.102	-.182	.016			-.063	.062	.093	-.227	.018			-.103	.063	.007	-.294	-.048		
Overtime Hours	.168	.005	<.001	.011	.030			.157	.006	<.001	.012	.034			-.092	.006	.021	-.025	-.002		
Job Changes	-.033	.021	.375	-.061	.023			-.003	.026	.932	-.054	.049			-.041	.027	.290	-.080	.024		
Household Income	-.006	.000	.886	.000	.000			.003	.000	.949	.000	.000			.079	.000	.050	.000	.001		
Marital Status	.034	.100	.445	-.120	.273			-.056	.123	.225	-.392	.092			.022	.124	.639	-.186	.303		
Presence of Minor Children	.108	.108	.014	.055	.478			.051	.133	.255	-.110	.413			-.057	.134	.210	-.432	.095		
Caregiving at Home	.178	.173	<.001	.502	1.183			.108	.214	.004	.193	1.034			-.017	.216	.660	-.519	.329		

*Note.* To account for multicollinearity, variables such as age, number of days off, and job characteristics (1. Need for communication with internal colleagues, 2. Need for communication with external parties, 3. Physical workload, 4. Mental stress, 5. Job complexity, and 6. Frequency of irregular tasks) were excluded as independent variables from the hierarchical multiple regression analysis.

*β* = standardized coefficient, *SE* = standard error.

**p < .01, *p < .05.

### Test-retest reliability

To evaluate test-retest reliability, a follow-up survey was administered three weeks after the initial survey. Of the 300 participants invited, 283 (169 males, 114 females, mean age = 47.39 years, SD = 12.26, range = 23–69 years) who met the IMC criteria were included in the analysis.

The intraclass correlation coefficients were calculated, resulting in values of  .749 for IWF,  .730 for WFS, and  .597 for WFE. These values indicated a satisfactory level of reliability, suggesting that the WLIS-J demonstrated consistent measurements over time.

## Discussion

### Development and reliability/validity of the WLIS-J

The primary aim of this study was to develop the WLIS-J, a culturally adapted version of the Work-Life Integration Scale suitable for the Japanese context. The analyses revealed a three-factor structure for Japanese WLI: Work–Family Strain (WFS), representing stress arising from role conflicts between work and family; Work–Family Enrichment (WFE), representing satisfaction derived from the integration of both domains; and Interference between Work and Family (IWF), representing the mutual interference between work and family roles.

While the original scale [11] identified a four-factor structure distinguishing between “work-to-family interference” and “family-to-work interference,” these two dimensions were integrated into a single construct in the WLIS-J, resulting in IWF as a unified factor. This difference likely reflects cultural characteristics of Japan, where the directionality of work–family interaction is less distinctly perceived.

In Japan’s collectivist culture, individuals tend to prioritize harmony within organizations and families over personal interests [26, 27]. Consequently, work and family are less likely to be viewed as separate domains and more likely to be recognized as complementary and continuous parts of one’s overall life. Moreover, Japan’s high uncertainty avoidance and long-term orientation [27] foster values that emphasize stability and harmony over time. As a result, maintaining an integrated balance between work and family is often regarded as ideal, and bidirectional interference between the two domains tends to be understood as a mutually influential experience rather than as a unidirectional conflict.

This cultural characteristic was further reflected in the concurrent validity of IWF. IWF showed positive correlations with both the Work–Life Conflict (WLC) scale and the K6 score, indicating associations with role conflict and psychological distress, while also correlating positively with WFE. Thus, IWF appears to encompass both conflict and satisfaction, suggesting a dual nature within the Japanese context. This seemingly paradoxical finding reflects the integrative and interactive nature of the relationship between work and family in Japan. In Japanese society, interpersonal relationships and a sense of responsibility in the workplace are closely tied to one’s identity [28], which facilitates the spillover of work-related experiences and emotions into family life, and vice versa. Within such a cultural context where role boundaries are blurred, the integration of work and family can generate both positive and negative effects. Therefore, the three-factor structure of the WLIS-J can be interpreted as reflecting the integrated and reciprocal experience of work and family among Japanese workers.

### Examination of the determinants of WLI

The second objective of this study was to identify the determinants of workers’ WLI. The results revealed that among individual, work-related, and family-related factors, work-related factors exerted the strongest influence and were significantly associated with all WLI subdimensions. In particular, longer working hours and overtime were associated with higher WFS and lower WFE, indicating that long working hours constitute a major factor that hinders the integration of work and family and increases psychological and physical burden. This finding is consistent with prior Western studies reporting that increased working hours exacerbate work–family conflict [13,14], suggesting that time-related constraints are also a fundamental barrier to WLI in Japan.

In contrast, teleworking hours were positively associated only with Interference between IWF and were not significantly related to WFS or WFE. In Western countries, teleworking has often been reported to facilitate flexible role adjustment and contribute to positive evaluations of work–life balance [29,30]; however, such tendencies were not observed in the present study. One possible explanation lies in the persistent “work-first” labor norms in Japan. Specifically, the intrusion of work into the family domain through teleworking may be less likely to be recognized as problematic and may be experienced not as autonomous integration but rather as passive integration driven by adaptation to organizational norms.

Regarding individual factors, men tended to report higher IWF, whereas women reported higher WFS. In Western contexts, boundary integration is often conceptualized as an expression of autonomous boundary management [29]. In Japan, however, where long working hours and strong organizational commitment are culturally expected [26,27], the intrusion of work into family life may emerge less as an autonomous choice and more as a form of normative adaptation. Thus, the higher IWF observed among men may reflect not flexibility but rather difficulty psychologically detaching from work even during family time. Meanwhile, the higher WFS observed among women may reflect the internalization of societal expectations surrounding family responsibilities in Japan.

Regarding family-related factors, the presence of a spouse showed no significant association, whereas the presence of children or home caregiving responsibilities was positively associated with both IWF and WFS. This suggests that it is not simply the presence of a partner, but rather concrete caregiving responsibilities such as childcare and eldercare, that increase interference with work and psychological burden. This finding is consistent with European research showing that conflicts between work and family caregiving responsibilities negatively affect workers’ mental health [31]. Furthermore, household income showed a weak positive association with WFE. This result is consistent with studies of Western workers indicating that economic stability functions as a buffer against work–life conflict [32] and supports work–family balance through increased life resources [33]. Other studies have shown that WFE is determined more strongly by workplace support and job autonomy than by family composition [34], which is also consistent with the present finding that the presence of children or caregiving responsibilities did not significantly affect WFE. Thus, as in Western contexts, economic resources in Japan may generate temporal and psychological flexibility that supports positive mutual enrichment between work and family.

Taken together, these findings reconfirm that time-related constraints at work are a major factor that disrupts the balance between work and family and intensifies workers’ conflict. Previous research has emphasized the burden created by multiple overlapping roles; however, in contemporary society, balancing multiple roles is often unavoidable, and the critical issue is how these roles are managed. From this perspective, traditional work–life balance (WLB), which assumes the separation of work and family domains, may paradoxically increase burden. In contrast, WLI represents a framework that emphasizes flexible integration and mutual adjustment between these domains, enabling reciprocal positive effects. WLI may therefore represent a novel approach that extends beyond conventional WLB.

At the same time, Japanese work culture is supported by cultural values that reward long working hours and prioritize work over family [4]. These cultural constraints function as structural barriers to WLI. Indeed, judging from the incremental R² values obtained in the present study, the influence of family-related factors was limited, whereas work-related factors exerted substantially greater influence on WLI. Therefore, improving WLI requires not only individual effort but also institutional reform at the organizational level. Specifically, organizations should promote performance-based evaluation systems and encourage flexible work arrangements through workload management and overtime reduction. Future research should also focus on qualitative investigations of workers who successfully achieve high WLI in order to clarify the facilitating factors specific to Japan. WLI provides a framework capable of addressing the increasingly diverse needs of workers, and its realization will require institutional reform and cultural transformation grounded in maximizing the synergistic relationship between work and life.

### Strengths and limitations

This study has two major contributions. First, it developed a Japanese version of the WLI scale. By adapting the scale to reflect Japan’s labor context, it has become possible to assess WLI among Japanese workers. Second, this study identified the factors that determine WLI, providing valuable insights into variables that will be critical for advancing future workstyle reform in Japan.

Nevertheless, several limitations should be acknowledged. First, the factor structure of WLI did not fully replicate previous findings. Although the WLIS-J demonstrated sufficient reliability and validity in the Japanese context, the identified three-factor structure differed from the original four-factor model. Therefore, the WLIS-J should be regarded not as a direct translation of the original scale but as a culturally adapted version. At present, the WLIS-J cannot be considered psychometrically equivalent to the original scale, and international comparisons using these measures should therefore be interpreted with caution. Second, this study employed a cross-sectional design, which limits causal interpretation. Third, although the sample broadly reflected the general population, certain age groups (e.g., individuals aged 50–59 years) were overrepresented, whereas others (e.g., individuals aged 20–29 years) were underrepresented. This imbalance may limit the generalizability of the findings. Future studies should improve representativeness through weighting procedures or stratified sampling methods. Fourth, criterion-related validity was not sufficiently examined in accordance with the COSMIN guidelines. In this study, self-reported work-related variables were included in the analysis to examine the validity of WLI within the Japanese labor context. However, these variables were not clearly predefined and operationalized as external criteria in the manner recommended by COSMIN and are therefore insufficient for formally evaluating criterion-related validity. Accordingly, although the present findings provide preliminary evidence for structural validity and construct validity, they cannot be considered sufficient evidence to fully support criterion-related validity. Future research should conduct a more rigorous evaluation of criterion-related validity in accordance with the COSMIN standards by clearly defining objective indicators obtainable from respondents’ organizations (e.g., working hours and productivity indicators) as external criteria and examining their associations with the scale.

## Conclusion

This study resulted in the successful development of the WLIS-J, and its reliability and validity were confirmed. Additionally, it clarifies which variables in employment conditions determine WLI.

## Supporting information

S1 Appendix Japanese version of the Work-life Integration Scale. (DOCX)

S2 Appendix Japanese version of the Work-life Integration Scale English Translation. (DOCX)

S1 Data Anonymized data set. (XLSX)
