# Supplementary material for: Development of the Japanese version of the work-life integration scale: Focusing on the work environment in Japan
Source: PLoS One. 2026 Sep 11;21(9):e0356695. doi: 10.1371/journal.pone.0356695 (PMC13568493; doi:10.1371/journal.pone.0356695)
Supplement: Supplementary file 1 [file pone.0356695.s001.docx]

Japanese version of the Work-life Integration Scale

以下を読み、1（全く当てはまらない）から7（最も当てはまる）のうち、該当するものを1つ選んでください。

Work-Life Integration Scale (Japanese version)

© 2025 Wakashima Laboratory, Tohoku University

This work is licensed under a Creative Commons Attribution 4.0 International License (CC BY 4.0).

https://creativecommons.org/licenses/by/4.0/
