# Supplementary material for: Development of the Japanese version of the work-life integration scale: Focusing on the work environment in Japan
Source: PLoS One. 2026 Sep 11;21(9):e0356695. doi: 10.1371/journal.pone.0356695 (PMC13568493; doi:10.1371/journal.pone.0356695)
Supplement: Supplementary file 2 [file pone.0356695.s002.docx]

Japanese version of the Work-life Integration Scale

Read the following sentences and choose the one that most applies to you from 1 (Applies the least) to 7 (Applies the most).

| 1 | While at work, I sometimes handle tasks concerning my family life. | 1 | 2 | 3 | 4 | 5 | 6 | 7 |
| --- | --- | --- | --- | --- | --- | --- | --- | --- |
| 2 | I worry about my family while I’m at work. | 1 | 2 | 3 | 4 | 5 | 6 | 7 |
| 3 | Family members contact me while I’m at work. | 1 | 2 | 3 | 4 | 5 | 6 | 7 |
| 4 | I have had to interrupt my work in the office many times to deal with family concerns. | 1 | 2 | 3 | 4 | 5 | 6 | 7 |
| 5 | I bring work home. | 1 | 2 | 3 | 4 | 5 | 6 | 7 |
| 6 | While at home, I’m constantly thinking about my workplace problems. | 1 | 2 | 3 | 4 | 5 | 6 | 7 |
| 7 | After returning home from work, I almost always answer work-related phone calls. | 1 | 2 | 3 | 4 | 5 | 6 | 7 |
| 8 | I interrupt my housework to solve work-related problems. | 1 | 2 | 3 | 4 | 5 | 6 | 7 |
| 9 | I feel like I'm taking on more work than I can handle. | 1 | 2 | 3 | 4 | 5 | 6 | 7 |
| 10 | When I return home from work, I feel that I’m physically worn out. | 1 | 2 | 3 | 4 | 5 | 6 | 7 |
| 11 | When I return home from work, I feel that I’m mentally worn out. | 1 | 2 | 3 | 4 | 5 | 6 | 7 |
| 12 | I feel that I’ve been unable to set aside enough time for myself. | 1 | 2 | 3 | 4 | 5 | 6 | 7 |
| 13 | To be able to handle work at the workplace and at home every day,  I feel that I have to be in a rush or keep myself busy all the time. | 1 | 2 | 3 | 4 | 5 | 6 | 7 |
| 14 | My work keeps me away from my family. | 1 | 2 | 3 | 4 | 5 | 6 | 7 |
| 15 | With my current job, I am able to balance my work and family life. | 1 | 2 | 3 | 4 | 5 | 6 | 7 |
| 16 | I have enough time for both my work and my personal life. | 1 | 2 | 3 | 4 | 5 | 6 | 7 |
| 17 | My current job schedule allows me to feel fulfilled. | 1 | 2 | 3 | 4 | 5 | 6 | 7 |
